# Stapedotomy or Stapedectomy: Does It Really Matter?

**DOI:** 10.1055/s-0044-1792086

**Published:** 2025-01-10

**Authors:** Francisco Teixeira-Marques, Rita Vaz Osório, Mónica Teixeira, Joana Rebelo, Sandra Gerós, Diamantino Helena, António Faria de Almeida, Pedro Oliveira

**Affiliations:** 1 Department of Otorhinolaryngology, Unidade Local de Saúde de Gaia e Espinho, Gaia, Portugal

**Keywords:** otosclerosis, stapedotomy, stapedectomy

## Abstract

**Introduction** Otosclerosis leads to stapes fixation and consequent conductive hearing loss. Surgery is the mainstay of treatment, and it can be achieved through small fenestra stapedotomy or stapedectomy. Despite the first being favored by most, evidence supporting its superiority over the latter remains inconclusive.

**Objective** To assess the hearing outcomes and complications of stapes surgery performed in a series of patients with otosclerosis and compare the results of stapedotomy with stapedectomy.

**Methods** A retrospective study of 134 ears in 125 otosclerosis patients undergoing primary stapes surgery was conducted. Patients underwent either stapedotomy or stapedectomy, and outcomes were compared using pre- and postoperative audiometric data and complication rates.

**Results** Most cases (81%) underwent stapedotomy. Both techniques resulted in significant improvement in air-bone gap (ABG) and speech recognition threshold (SRT) postoperatively, with no significant difference between them. Complication rates were comparable between techniques, with no statistical difference in postoperative complications.

**Conclusion** Both stapedotomy and stapedectomy offer favorable hearing outcomes with low complication rates in otosclerosis patients. While stapedotomy remains the preferred technique, if the initial plan shifts to a stapedectomy, surgeons should remain composed and confident in a good hearing outcome.

## Introduction

Otosclerosis is a condition caused by abnormal bone remodeling in the optic capsule that leads to stapes fixation and consequent conductive hearing loss. Diagnosis is presumed by the presence of conductive hearing impairment in patients with normal external auditory canal and tympanic membrane, and computed tomography (CT) scan findings of abnormal bone densities within the optic capsule. 1 Stapes surgery is the gold standard treatment for otosclerosis and aims to restore the sound transmission mechanism by replacing the stapes with a prosthetic device that allows the sound waves to travel to the inner ear.

Since the first stapedectomy performed by John Shea in 1956, stapes surgery has evolved, and many variations of the technique have been published. 2 The major innovation was the replacement of stapedectomy (total removal of the footplate) for stapedotomy (small fenestra in the footplate just large enough to accommodate the piston) as the preferred technique by most otologists, due to the belief that stapedotomy provides: i) better hearing improvement, ii) more stable long-term hearing results, iii) lower incidence of complications (sensorineural hearing loss, postoperative vertigo, perilymphatic fistula). 1 Because of this perception, most ear surgeons opt for stapedotomy whenever possible. 3 However, these differences remain debatable, with many studies reporting similar results between the two techniques. 4 5 6 7

The authors have two main purposes with this study: i) to assess the hearing outcomes and complications of stapes surgery performed in a series of patients with otosclerosis; and ii) to compare the results of stapedotomy with stapedectomy performed in our otorhinolaryngology department.

## Methods

We conducted a retrospective study of 142 ears in 125 patients with otosclerosis that underwent stapes surgery in the otorhinolaryngology department of a tertiary level hospital in the last 10 years (2014–2023). Revision surgeries (n = 8) were excluded from the study. Medical records were investigated for data collection regarding demographic information, surgical report, audiometric results, and postoperative complications. The last preoperative audiogram before surgery and the 6 months postoperative audiogram were used. The air-bone gap (ABG) was calculated from the difference between the mean air- and bone-conduction thresholds obtained at 0.5, 1, 2, and 4 kHz. The speech recognition threshold (SRT) was defined as the minimum hearing level for speech at which an individual can recognize 50% of the information, and was measured in the pre- and postoperative hearing evaluations.

Every surgery was performed under general anesthesia. After confirmation of a stapedial fixation, a hole was made in the footplate with a perforator to perform stapedotomy. On the other hand, stapedectomy was performed only when the footplate accidentally cracked or was removed along with the stapes superstructure. In stapedectomy cases, the decision to use a tissue seal of the fenestra (tragus perichondrium or temporalis fascia graft) or not was made by the surgeon. A Teflon prosthesis with 0.6 mm in width was used in all cases.

The ears were divided into the stapedotomy and stapedectomy groups according to the chosen surgery technique. Hearing outcomes and surgical complications were analyzed and compared between the groups. Patients who underwent stapedotomy in one ear and stapedectomy in the other were placed in a subgroup in order to compare surgical outcomes between them.

Statistical analysis was performed using the IBM SPSS Statistics for Windows (IBM Corp., Armonk, NY, USA) software, version 27.0. The nonparametric Mann-Whitney U test was used to compare hearing outcomes and complication between the stapedotomy and the stapedectomy groups. Values of *p* < 0.05 were considered statistically significant.

## Results

During the study period, 134 ears from 125 patients were deemed eligible based on the inclusion and exclusion criteria (revision surgeries from 6 stapedectomies and 2 stapedotomies were excluded). Among the 134 cases analyzed, 109 (81%) underwent stapedotomy, while 25 (19%) underwent stapedectomy. The mean age of the surgical patients was of 47.3 ± 10.8 years, with 34% male and 66% female subjects. Table 1 presents demographic data for patients in the stapedotomy and stapedectomy groups, excluding revision cases. There were no statistically significant differences ( *p* > 0.05) between the groups regarding age, gender, laterality, type of hearing loss or length of hospital stay.

**Table 1:** Demographic data of the study population

*Demographic data*	*Stapedotomy* *n* *=* *109*	*Stapedectomy* *n* *=* *25*
***Mean age*** **,** ***years***	47.4 ± 8.8	48.3 ± 8.6
***Gender***		
* Male*	39 (35.8%)	6 (24.0%)
* Female*	70 (64.2%)	19 (76.0%)
***Both ears affected***	63 (57.8%)	17 (68.0%)
***Type of hearing loss***		
* Conductive*	38 (34.9%)	12 (48.0%)
* Mixed*	71 (65.1%)	13 (52.0%)
***Side of surgery***		
* Right*	58 (53.2%)	15 (60.0%)
* Left*	51 (46.8%)	10 (40.0%)
***Days of hospitalization***	2.36 ± 0.86	2.40 ± 0.85

The length and diameter of prostheses used in both patient groups were recorded and compared ( Table 2 ). Teflon pistons with a width of 0.6 mm and lengths ranging from 4 to 5 mm were selected based on the distance between the incus process and stapes footplate measured during surgery. In stapedectomy surgeries, most surgeons used tragal perichondrium to seal the oval window (n = 16; 64.0%), while only 1 (4.0%) used temporal fascia, and 8 (32.0%) did not use any additional tissue besides the prostheses.

**Table 2:** Prostheses used in each surgical procedure

*Prostheses* *(widthxlenght in mm)*	*Stapedotomy* *n = 109*	*Stapedectomy* *n = 25*
*0.6 × 4*	6 (5.5%)	2 (8.0%)
*0.6 × 4.25*	26 (23.9%)	7 (28.0%)
*0.6 × 4.5*	65 (59.6%)	14 (56.0%)
*0.6 × 4.75*	12 (11.0%)	1 (4.0%)
*0.6 × 5*	0 (0%)	1 (4.0%)

**Fig. 1:**
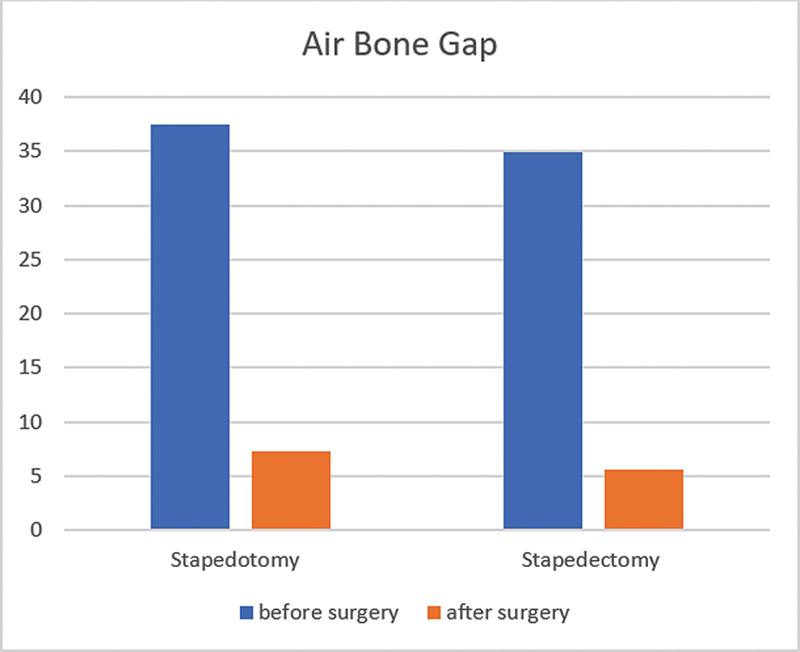
Differences in pre- and postoperative air-bone gap in both groups.

The presurgical ABG in patients who underwent stapedotomy was of 37.42 ± 8.63, and the postsurgical ABG was of 7.27 ± 13.64, with a *p* -value < 0.001. For patients who underwent stapedectomy, the pre-surgical ABG was of 34.88 ± 7.84, and the postsurgical ABG was of 5.54 ± 9.88, also with a *p* -value < 0.001 ( Fig. 1 ). Pre- and postsurgical ABG between these 2 groups were not statistically significant ( *p* : 0.533 and 0.196 respectively).

**Fig. 2:**
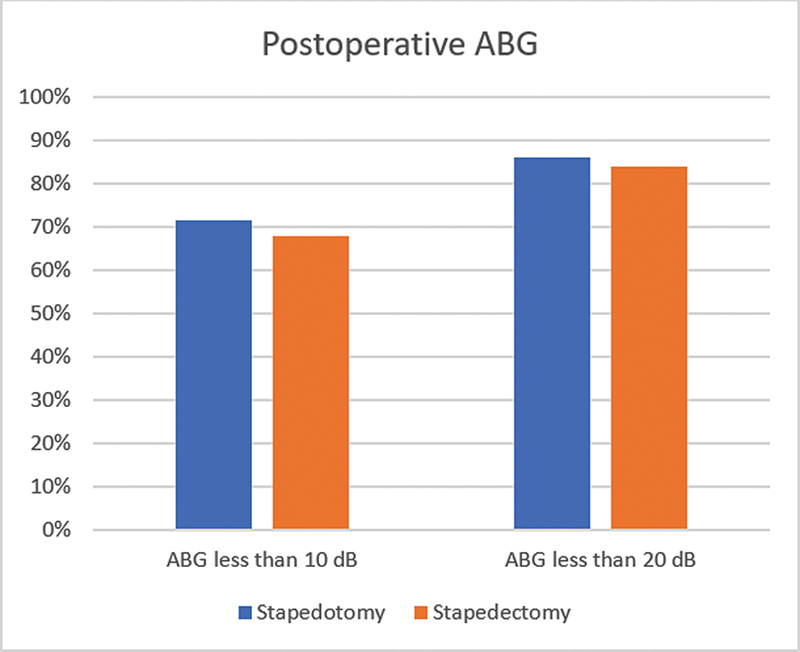
Postoperative air-bone gap < 10 and < 20 dB in both groups.

The postoperative ABG was evaluated to determine surgery success. In the stapedotomy group, 78 (71.6%) patients achieved an ABG below 10 dB, and 94 (86.2%) achieved an ABG below 20 dB. In the stapedectomy group, 17 (68.0%) patients achieved an ABG below 10 dB, and 21 (84.0%) achieved an ABG below 20 dB. There were no statistically significant differences between the 2 groups ( *p* = 0.745). These postoperative results are depicted in Figure 2 .

**Fig. 3:**
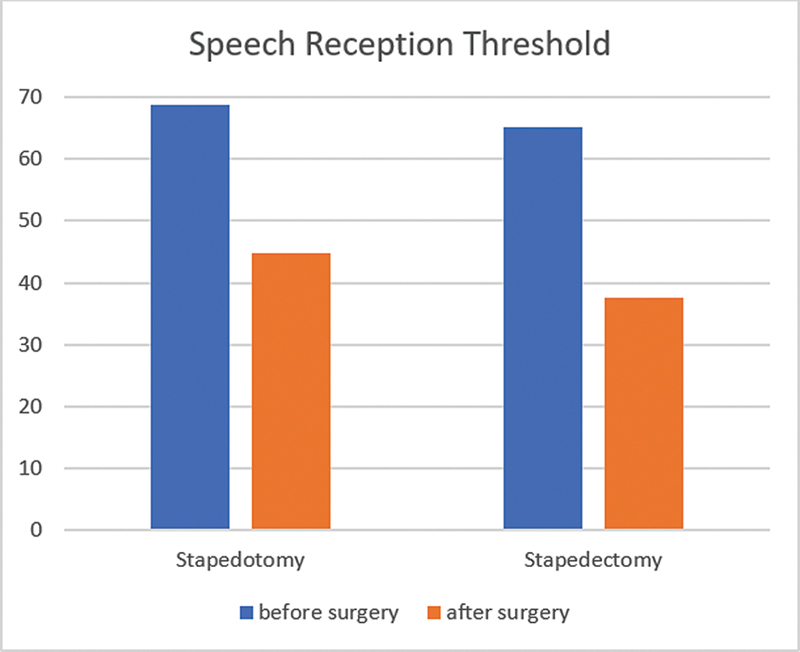
Speech reception threshold pre- and postsurgery in both groups.

The SRT before (68.67 ± 13.48) and after (44.88 ± 16.49) stapedotomy showed statistical significance ( *p* < 0.001). Similarly, for stapedectomy, the SRT before (65.20 ± 15.17) and after (37.61 ± 11.37) surgery was statistically significant ( *p* < 0.001), as shown in Fig. 3 . There was no statistically significant difference in terms of SRT between the two groups ( *p* : 0.509 and 0.077 respectively).

There was no significant difference in the percentage of patients with ABG closure within 10 ( *p* = 0.726) or 20 dB ( *p* = 0.556), indicating no discernible differences in postoperative hearing outcomes between the two surgical techniques.

Additionally, five patients underwent stapedotomy in one ear and stapedectomy in the other, with all ten ears achieving a postoperative ABG below 10 dB.

Concerning postoperative complications, 1 patient experienced labyrinthitis after stapedotomy, while 3 patients in the stapedotomy group (2.7%) and 1 in the stapedectomy group (4%) suffered from profound sensorineural hearing loss (2 patients in the first week, 1 patient 4 weeks after surgery, 1 patient 6 months after surgery). Additionally, symptoms of vertigo were present at 6 months postoperatively in 11 poststapedotomy (10.1%) and 4 poststapedectomy (16%) cases. Statistical analysis revealed no significant differences in postoperative complications between the surgical techniques ( *p* = 0.911), as summarized in Table 3 .

**Table 3:** Postoperative complications

*Postoperative complications*	*Stapedotomy* *n = 109*	*Stapedectomy* *n = 25*
*Revision surgery*	6 (5.2%)	2 (7.4%)
*Severe sensorineural hearing loss*	3 (2.7%)	1 (4%)
*Labyrinthitis*	1 (0.9%)	0 (0%)
*Vertigo at 6 months postop* eratively	11 (10.1%)	4 (16%)

## Discussion

Conductive hearing loss caused by otosclerosis can be managed by two different approaches: hearing aids or stapes surgery. Nevertheless, stapes surgery is the only treatment option for otosclerosis that can restore sound transmission, being recognized as more effective and with better quality of life improvement for the patient. 8 Currently, stapedotomy is the preferred surgical technique because of the greater improvements in hearing at higher frequencies shown with this technique, as well as the perception of lower complication rates. 8 However, it is believed that the surgeons' experience plays the most important part in the success of stapes surgery. 7

Most patients in our cohort underwent stapedotomy. The reason for this discrepancy is that, in our department, that is our standard, and stapedectomy is performed only when it is not doable, either because the footplate accidentally cracks, or is removed along with the stapes superstructure. In the present study we focused on comparing both techniques regarding postoperative hearing results and complications of our department, in order to enhance the knowledge surrounding stapes surgery.

Regarding hearing outcomes, both stapedotomy and stapedectomy demonstrated good results and no differences, corroborating previous reports of similar studies. 4 5 6 7 The piston used in all surgeries was Teflon, with 0.6 mm in diameter, while the length of the prosthesis differed according to intraoperative findings. We did not calculate the difference of hearing improvement between each frequency because it is believed that, although stapedotomy offers better results in higher frequencies, it does not offer clinical significance in terms of hearing function. 4 Disparate reports have been published in the literature. Some advocate greater hearing outcomes and less complications with stapedotomy, 9 10 11 and others note no differences in postoperative results between either technique. 4 5 6 7 12 Our results support the latter, with both our groups showing similar hearing results in postoperative ABG and SRT.

Additionally, as House et al. 4 had examined, we compared 5 patients who had undergone stapedotomy in one ear and stapedectomy in the other, allowing for a paired case comparison, and found complete closure of the ABG in each of the 10 ears with no complications reported, highlighting the efficacy of both techniques in ears with the same individual characteristics.

Complication rates have been reported as higher in patients that underwent stapedectomy over stapedotomy. 13 That was not the case in our cohort, but the small sample of complications in both techniques prevented us from establishing a causal correlation. Postoperative vertigo is common following stapes surgery and is the main reason for prolonged hospital stay, despite usually being resolved with conservative management. 14 In the present study we did not find a difference in days of hospitalization between the two groups, meaning the extent of the fenestra in the footplate had no association with the intensity of immediate postoperative vertigo. Furthermore, when comparing reported vertigo at 6 months postsurgery, we found no difference between groups, which was in accordance with the findings of Harmat et al.. 15

One of the patients with prolonged vertigo after stapedectomy underwent revision surgery despite having a postoperative ABG below 10 db, because there was a suspicion that the prosthesis was too long (0.6 mm in length), as reported by Job et al. 16 The piston was switched for a 0.45-mm one, with complete resolution of vertigo, maintaining a successful hearing outcome.

The most feared complication of stapes surgery is severe sensorineural hearing loss, a known rare complication that can occur in 0 to 11% of cases. 1 17 In our cohort, we found 4 cases (stapedotomy: 3; stapedectomy: 2), either early or late in the postoperative period. In one of those, revision surgery was performed and the prosthesis removed, with no success. This overall rate of 3% is significant, with two possible explanations: 1) small sample number in comparison with other reports; 2) the different surgeons who operated in our cohort, corroborating that experience may be the most important determinant of success in stapes surgery. 18

## Conclusion

In the present study, we were able to show that both stapedotomy and stapedectomy offer good hearing results with a low percentage of complications in patients with otosclerosis. Although stapedotomy remains the preferred technique for most ear surgeons, one should note that if the initial plan shifts to a stapedectomy, surgeons should remain composed and confident in a good hearing outcome.
